# Supplementary material for: Metformin Influence on the Intestinal Microbiota and Organism of Rats with Metabolic Syndrome
Source: Int J Mol Sci. 2022 Jun 20;23(12):6837. doi: 10.3390/ijms23126837 (PMC9224185; doi:10.3390/ijms23126837)
Supplement: Supplementary file 1 [file ijms-23-06837-s001.zip › ijms-1725717-supplementary.pdf]

International Journal of
Molecular Sciences

MDPI

Supplementary Materials

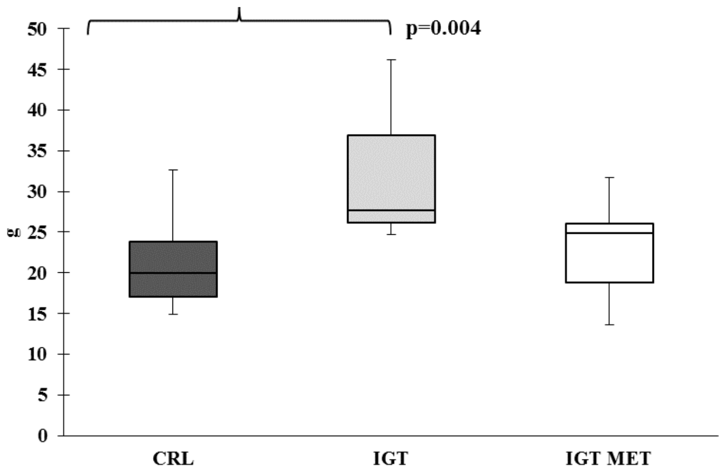

Figure S1. The omentum weight of rats from different groups at the end of experiment. Results are presented as median (25%; 75%). Notes: CRL – control group, IGT – impaired glucose tolerance group, IGT MET – impaired glucose tolerance + metformin group.

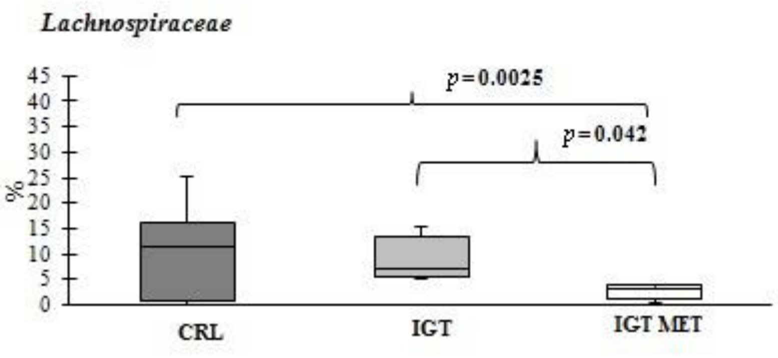

Figure S2. Relative abundances of family *Lachnospiraceae* of fecal samples of rats from differentups. Results are presented as median (25%; 75%). Notes: CRL – control group, IGT – impaired glucose tolerance group, IGT MET – impaired glucose tolerance + metformin group.

Table S1. The changes revealed after impaired glucose tolerance induction.

Parameters	Increase	Decrease
Clinic and laboratory parameters	Weight of rats<br>Weight of omentum<br>Glucose level<br>Total cholesterol<br>Atherogenic coefficient<br>Ischemia	Functional of myocardium

qPCR (quantity)	*Acinetobacter* spp.	*Lactobacillus* spp.
Metagenome 16S rRNA study (relative abundance)	*Firmicutes phylum*<br>*Akkermansia* spp.<br>*Bacteroides* spp.<br>*Roseburia*spp.	

Table S2. The changes revealed after metformin treatment of rats.

Parameters	Increase	Decrease
Clinic and laboratory parameters		Body weight of rats<br>Glucose level<br>Cholesterol<br>Cardial infarct sise
qPCR (quantity)	*Akkermansia muciniphila*<br>*Lactobacillus* spp.	*Acinetobacter* spp.<br>*Roseburia unulinivorans*
Metagenome 16S rRNA study (relative abundance)	*Actinobacteria phylum*<br>*Akkermansia* spp.	*Firmicutes phylum*<br>*Lachnospiraceae family*<br>*Roseburia*spp.<br>*Bacteroides* spp.

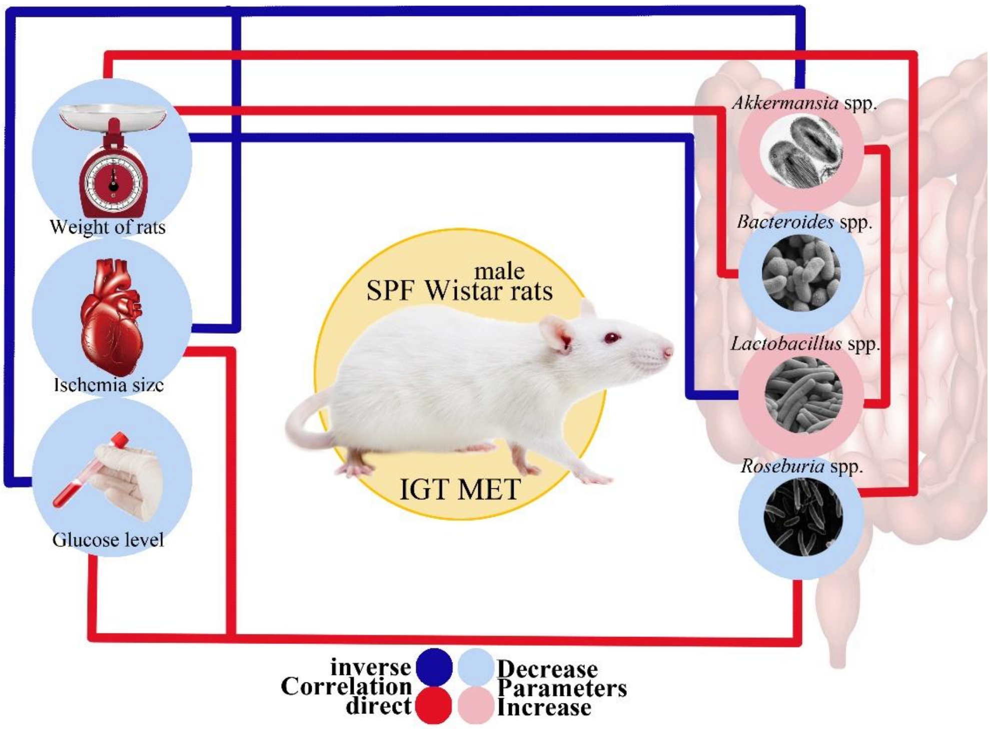

Figure S3. Correlations between the studied parameters after metformin therapy.

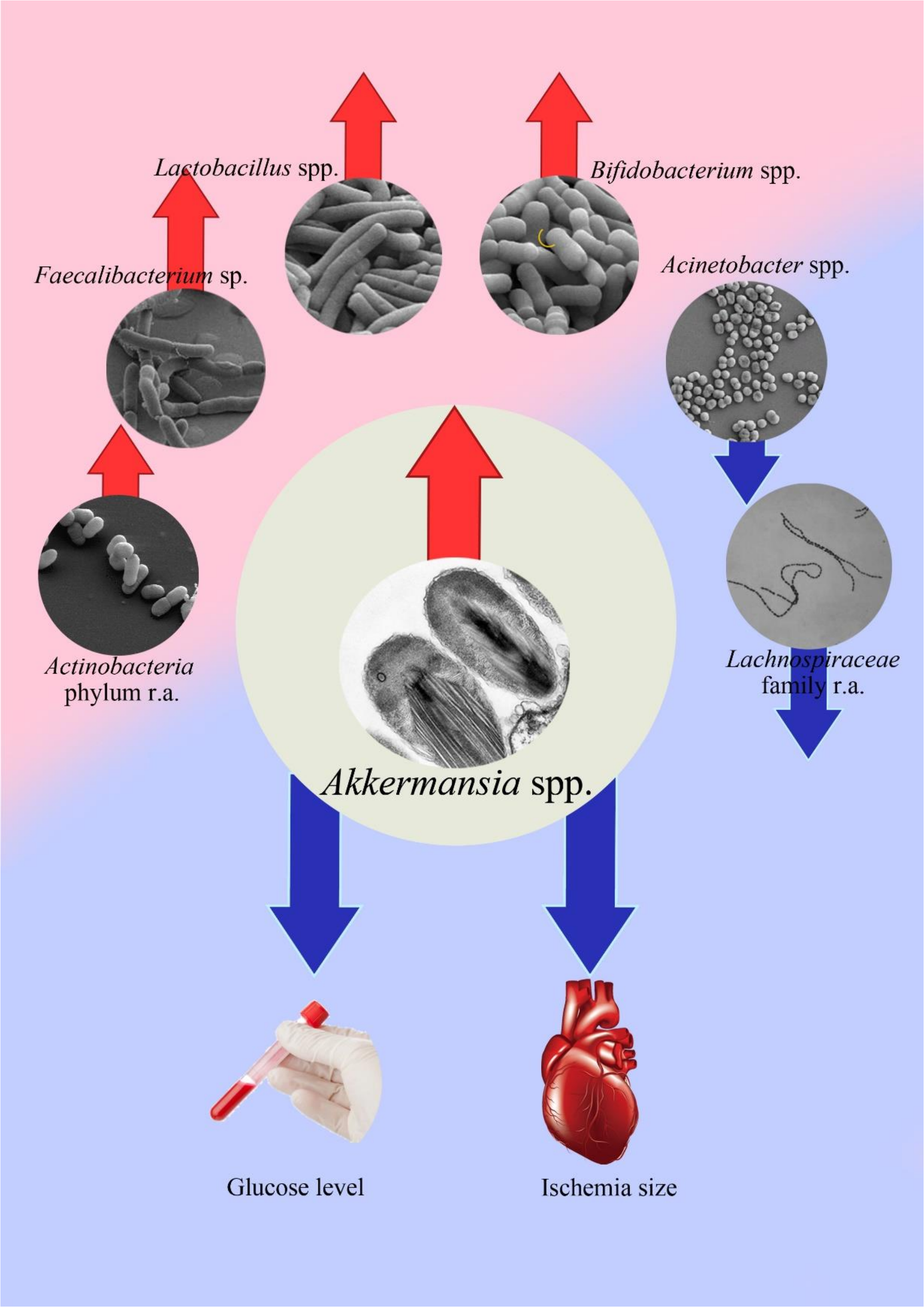

Figures S4. Changes of akkermansia representation and other parameters, connected with this bacteria.

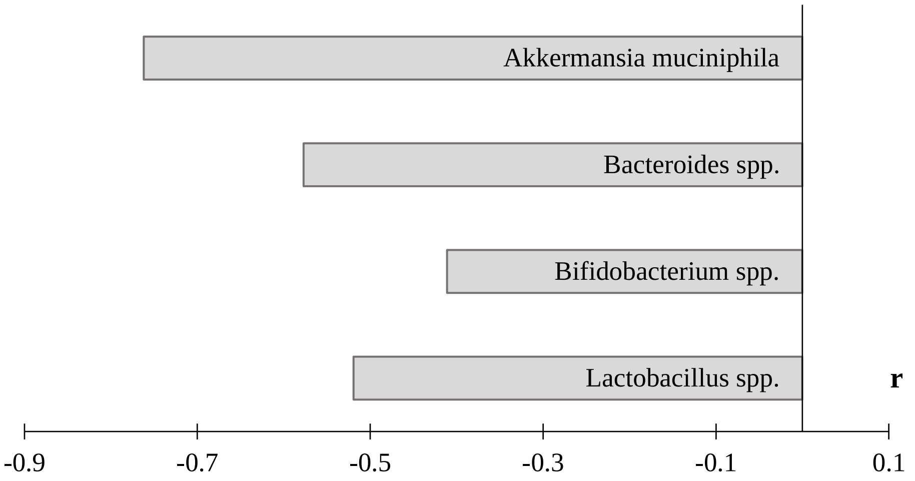

Figure S5. Coefficients of correlation by analysis between quantitative content of *Acinetobacter* spp. and other taxa in the intestinal microbiota at the end of the experiment. Notes: total data for all animals, the results of the study are presented using $p < 0.05$. Search for correlations between the studied parameters was performed using Spearman’s test.

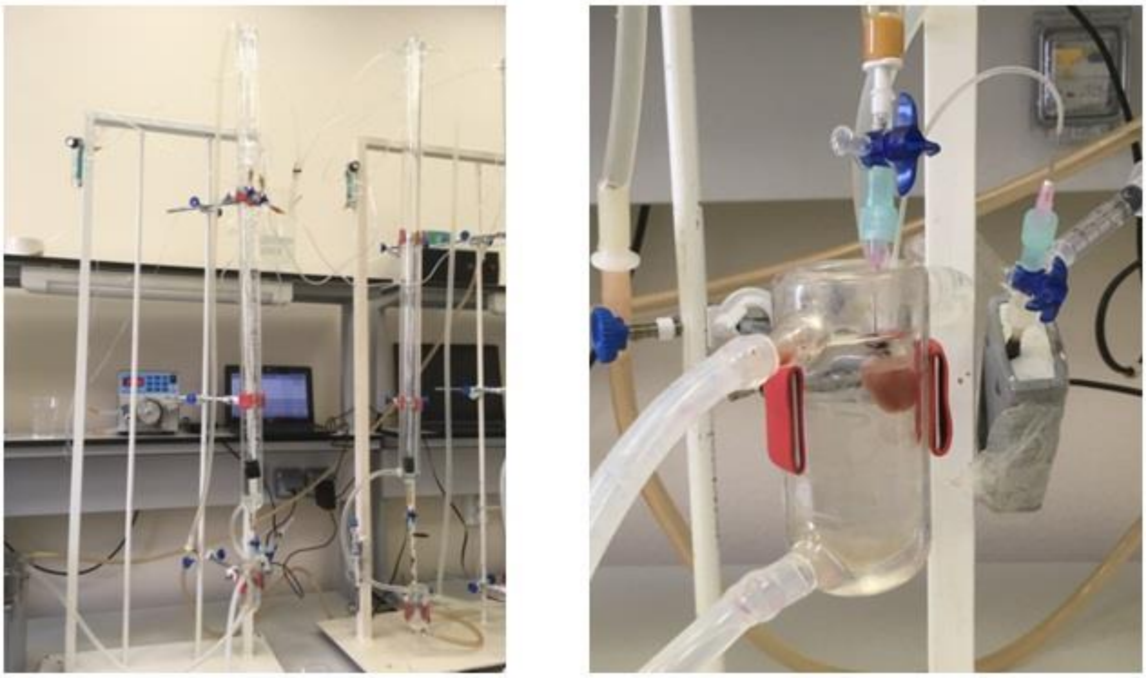

A **B**

Figure S6. Isolated heart perfusion according to Langendorff. (**A**) modified Langendorff apparatus. (**B**) isolated perfusion of rat heart by Langendorf.
